# The Rab11 effectors Fip5 and Fip1 regulate zebrafish intestinal development

**DOI:** 10.1242/bio.055822

**Published:** 2020-10-23

**Authors:** Cayla E. Jewett, Bruce H. Appel, Rytis Prekeris

**Affiliations:** 1 Department of Cell and Developmental Biology, University of Colorado School of Medicine, Aurora, CO 80045, USA; 2 Department of Pediatrics, University of Colorado School of Medicine, Aurora, CO 80045, USA

**Keywords:** FIP5, FIP1, Rip11, RCP, Rab11, Microvilli

## Abstract

The Rab11 apical recycling endosome pathway is a well-established regulator of polarity and lumen formation; however, Rab11-vesicular trafficking also directs a diverse array of other cellular processes, raising the question of how Rab11 vesicles achieve specificity in space, time and content of cargo delivery. In part, this specificity is achieved through effector proteins, yet the role of Rab11 effector proteins *in vivo* remains vague. Here, we use CRISPR/Cas9 gene editing to study the role of the Rab11 effector Fip5 during zebrafish intestinal development. Zebrafish contain two paralogous genes, *fip5a* and *fip5b*, that are orthologs of human *FIP5*. We find that *fip5a*- and *fip5b*-mutant fish show phenotypes characteristic of microvillus inclusion disease, including microvilli defects and lysosomal accumulation. Single and double mutant analyses suggest that *fip5a* and *fip5b* function in parallel and regulate trafficking pathways required for assembly of keratin at the terminal web. Remarkably, in some genetic backgrounds, the absence of Fip5 triggers protein upregulation of a closely related family member, Fip1. This compensation mechanism occurs both during zebrafish intestinal development and in tissue culture models of lumenogenesis. In conclusion, our data implicate the Rab11 effectors Fip5 and Fip1 in a trafficking pathway required for apical microvilli formation.

## Abstract (precis)

**Summary:** The Rab11 effectors *fip5a* and *fip5b* function in parallel to regulate apical trafficking and microvilli establishment during zebrafish intestinal development. Moreover, cells upregulate Fip1 to compensate for loss of Fip5.

## INTRODUCTION

Development of many organs, such as the gastrointestinal system, kidneys and respiratory tract, requires morphogenetic remodeling of cells to form a hollow tube, or lumen (Jewett and Prekeris, 2018). Whereas the mechanisms cells use to form a lumen vary by organ, a common feature is that cells adopt a highly polarized conformation including establishment of apical structures such as primary cilia, motile cilia or microvilli (Apodaca and Gallo, 2013). Intestinal epithelia are one of the few vertebrate cell types to lack primary cilia, but their apical cell surface is covered with a brush border composed of actin-rich membrane protrusions called microvilli to aid in nutrient absorption (Apodaca and Gallo, 2013). The molecular basis of cell polarization is well defined, but much less is understood about how trafficking pathways govern formation of these apical structures, especially *in vivo*.

The Rab11 apical recycling endosome pathway is a well-established regulator of polarity and lumen formation (Jewett and Prekeris, 2018). However, Rab11-directed trafficking events are also implicated in a number of other cellular processes, raising the question of how Rab11 vesicles achieve specificity when involved in numerous cellular functions. In part, Rab specificity is achieved through interaction with effector proteins, and Rab11 in particular interacts with a family of effector proteins called Rab11-family interacting proteins (FIPs) (Horgan and McCaffrey, 2009). There are five FIP family members, all of which contain a coiled-coil region at the C-terminus of the protein, allowing dimerization and binding to two Rab11 molecules, effectively forming a functional heterotetramer. Different FIPs appear to function in unique cellular processes including cytokinesis (FIP3 and FIP4), ciliogenesis (FIP3) and cargo recycling to the cell surface (FIP1, FIP2 and FIP5) (Horgan and McCaffrey, 2009). Previously, our lab implicated FIP5 in apical lumen formation in 3D Madin Darby Canine Kidney (MDCK) cell culture. We and others have shown that Rab11-FIP5 endosomes are required for lumenogenesis and interact with the actin-binding protein MYO5B to traffic cargo to the apical cell surface (Lapierre et al., 2001; Willenborg et al., 2011; Mangan et al., 2016). However, whether FIP5 plays a role in coordinating lumen morphogenesis during development *in vivo* is unknown.

Polarization is critical for cell function such that polarity disruption results in a number of diseases. Microvillus Inclusion Disease (MVID) is one such example, arising from the inability to form and maintain microvilli at the apical cell surface (Al-Daraji et al., 2010). Patients with MVID suffer from intractable diarrhea and malabsorption due to absent or very sparse microvilli and typically do not live past childhood. At the cellular level, patients display characteristic trafficking defects of lysosome accumulation and microvillus inclusions (Phillips et al., 1985; Phillips and Schmitz, 1992; Ruemmele et al., 2006). Mutations in MYO5B are found in patients with MVID and mutations in the zebrafish ortholog *myoVb* (also called *goosepimples*) and result in microvillus inclusions and trafficking defects (Müller et al., 2008; Ruemmele et al., 2010; Sidhaye et al., 2016). Moreover, experiments from intestinal tissue culture models suggest that the interaction between Rab11 and MYO5B is essential for microvilli maintenance (Knowles et al., 2014). Given that FIP5 interacts with MYO5B and is required for lumen formation in tissue culture, we hypothesized that FIP5 regulates intestinal development and microvilli formation *in vivo*.

## RESULTS

The mechanisms by which cells polarize and form an apical lumen have been studied extensively in 3D tissue culture, but whether these processes are recapitulated *in vivo* is unclear, because vertebrate models are inherently more complex and have compensatory mechanisms. Furthermore, intestinal tissue culture models are limited due to a lack of proper microvilli that are subject to the stresses and strains encountered by a functional animal intestine. To address these limitations, we used zebrafish intestinal development as an *in vivo* model of lumenogenesis and microvilli formation. We first examined the degree to which zebrafish Fip5 protein was conserved with human and dog FIP5 protein, as most work on FIP5 during cell polarization has been performed in MDCK cells. Zebrafish contain Fip5a and Fip5b orthologs to mammalian FIP5 with two highly conserved functional domains: a phospholipid-binding domain C2 domain at the N-terminus and a coiled-coil region at the C-terminus of the protein (Fig. S1A, yellow and blue highlight, respectively) required for dimerization and binding to Rab11 (Prekeris et al., 2001). Zebrafish intestinal development begins around 3 days post-fertilization (dpf) when many small lumens develop throughout the intestinal tract and subsequently fuse to form a single continuous lumen from mouth to anus (Ng et al., 2005; Alvers et al., 2014). To determine where *fip5a* and *fip5b* were expressed in zebrafish larvae during development, we performed *in situ* hybridization on 4 dpf larvae. Luminal organs such as the intestine, spinal cord and notochord expressed *fip5a* and *fip5b* (Fig. S2A,B). In measuring mRNA levels of *fip5a* and *fip5b*, we found that both transcripts showed increased levels around 3 dpf, and high levels of *fip5b* mRNA persisted throughout 8 dpf (Fig. S2C). We thus focused our efforts first on *fip5b*.

### Zebrafish intestinal microvilli establishment requires Fip5b

**Fig. 1.**
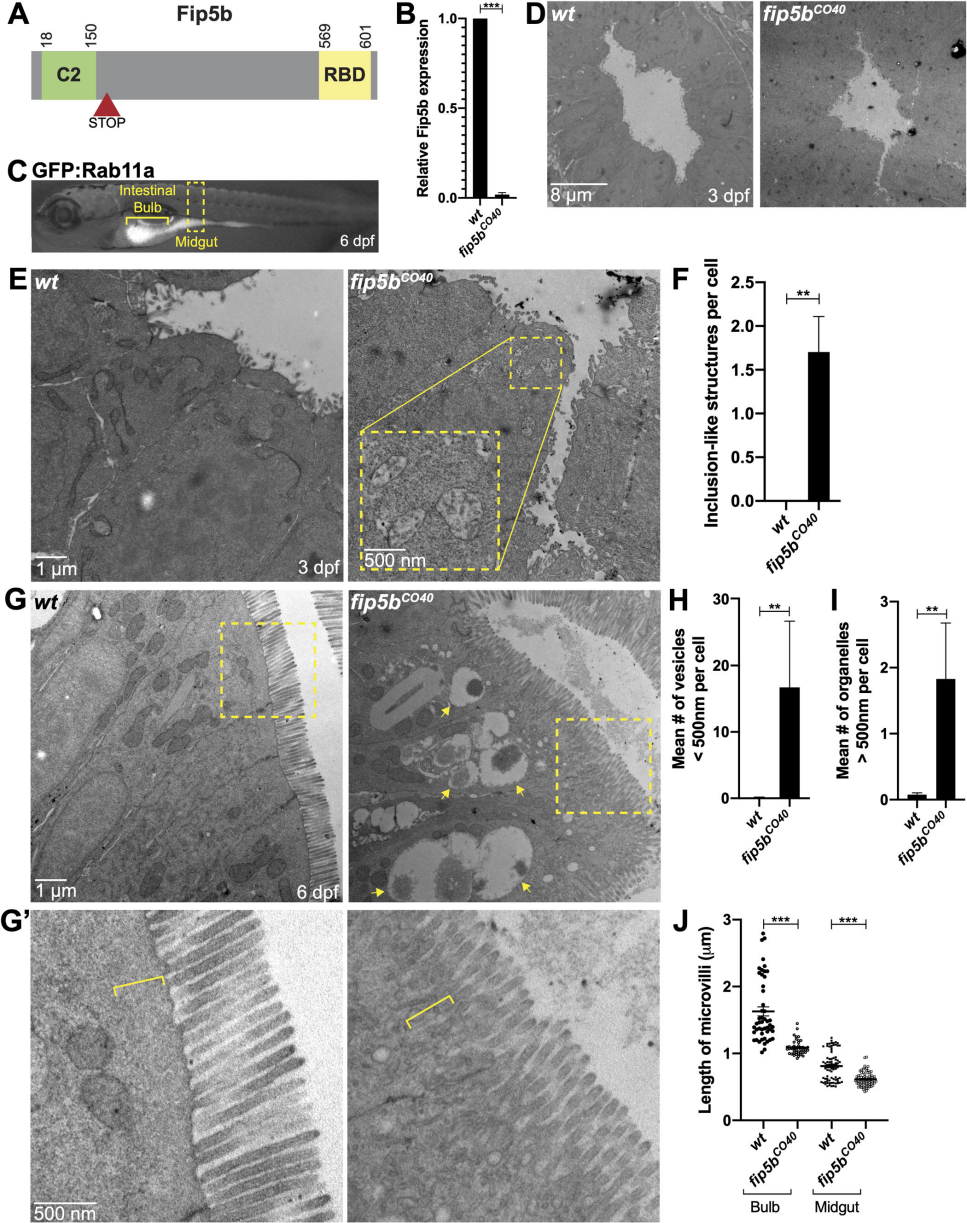
**Zebrafish intestinal microvilli establishment requires Fip5b.** (A) Domain schematic of zebrafish Fip5b protein containing a C2 domain at the N-terminus and a Rab-binding domain (RBD) at the C-terminus. The red arrowhead with STOP denotes premature termination codon in *fip5b* mutant alleles. (B) qRT-PCR for *fip5b* in wild-type and *fip5b^CO40^* mutant larvae at 6 dpf. *n*=50 larvae for each condition. (C) 6 dpf larvae expressing *Tg(hsp:GFP:Rab11a)* labeling the intestine. The intestinal bulb is denoted by a bracket and the midgut by a dashed box. All following images are representative cross sections through the midgut region. Wild-type siblings are used as controls. For all experiments, three separate animals for each condition were analyzed. (D) Electron micrographs showing 3 dpf wild-type and *fip5b^CO40^* mutant larvae. Luminal space is lighter gray region. (E) High magnification electron micrographs showing 3 dpf wild-type and *fip5b^CO40^* mutant larvae. Yellow box shows zoomed in view on a region with subapical microvillus inclusion-like structures. (F) Quantitation of the mean number of inclusion-like structures per cell in 3 dpf wild-type and *fip5b^CO40^* mutant larvae. (G) Electron micrographs showing 6 dpf wild-type and *fip5b^CO40^* mutant larvae. Arrows point to larger than 500 nm organelles and boxes outline magnified region shown in (G′). (G′) Magnified view of apical cell surface showing terminal web and microvilli. Brackets denote electron dense line and organelle-free zone comprising the terminal web or lack thereof in mutants. (H) Quantitation of less than 500 nm apical vesicles in 6 dpf larvae. (I) Quantitation of greater than 500 nm organelles in 6 dpf larvae. (J) Quantitation of microvilli length in the intestinal bulb and midgut in 6 dpf larvae. Each dot represents a single microvillus length combined across three animals. All plots show mean±s.e.m. A *t*-test was used for Gaussian data and a Mann–Whitney test for all other statistics. ***P<0.0005, **P<0.005.

To study the function of Fip5b, we used CRISPR/Cas9 gene editing. We selected two different *fip5b* alleles that introduced a premature stop codon right after the C2 domain at the N-terminus (Fig. 1A; Fig. S1B), thereby eliminating the Rab-binding domain (RBD) at the C-terminus essential for Fip5 function. We maintained these *fip5b* mutant stocks in a heterozygous state and performed intercrosses to generate zygotic mutants for analysis. Stage matched wild-type siblings were used as controls. We performed qRT-PCR to measure *fip5b* expression in *fip5b^CO40^*-homozygous mutant larvae and observed an almost complete loss of *fip5b* mRNA levels (Fig. 1B), suggestive of nonsense-mediated decay. *fip5b^CO40^*-homozygous mutant fish appeared morphologically normal from embryo through adulthood and were homozygous viable as adults. However, to determine if loss of Fip5b affected intestinal development at the cellular level, we performed transmission electron microscopy on fixed sections through the midgut region (Fig. 1C, yellow box) at developmental time points. At 3 dpf, when intestinal lumen morphogenesis initiated, *fip5b^CO40^* mutant larvae formed a single lumen (Fig. 1D), but upon closer examination, we noticed an accumulation of membrane vesicles in the subapical cytoplasm not present in wild-type larvae (Fig. 1E yellow box, F). These structures resembled microvillus inclusions, which are pathological hallmarks of MVID. At 6 dpf when intestinal development was mostly complete (Ng et al., 2005), microvillus inclusion-like structures were no longer evident near the subapical surface. Instead, intestinal cells of homozygous mutant larvae showed an accumulation of small (less than 500 nm) apical vesicles (Fig. 1G,H) and large (greater than 500 nm) organelles that resided medially in the cells (Fig. 1G arrows, I) compared to wild-type cells which did not show an accumulation of intracellular vesicles. Moreover, microvilli were shorter in both the anterior intestinal bulb and posterior midgut of 6 dpf homozygous mutant fish compared to wild-type siblings (Fig. 1G,G′,J). Finally, the terminal web, an apical cytoskeletal network anchoring microvilli into the cell, was disrupted in mutant fish. Wild-type larvae had a defined, electron-dense line at the base of the microvilli and an organelle-free zone just below the apical cell surface which was absent in mutants (Fig. 1G, boxed region magnified in G′, bracket). These data revealed trafficking and microvilli defects in *fip5b^CO40^*-mutant larvae.

### Endosome maturation and terminal web keratin organization require Fip5b function

**Fig. 2.**
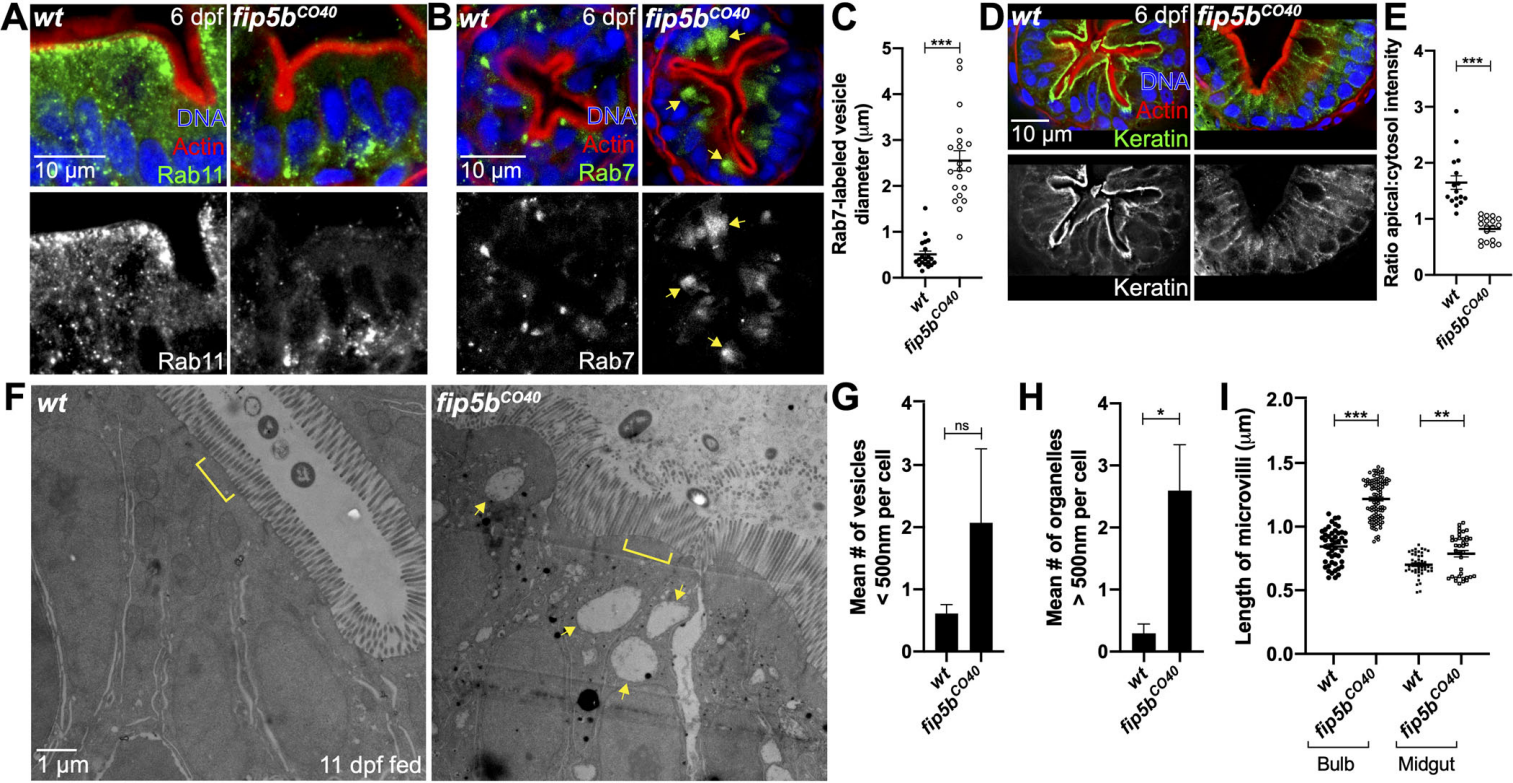
**Endosome maturation and terminal web keratin organization require** ***fip5b*** **function.** All following images are representative cross sections through the midgut region. Wild-type siblings are used as controls. For all experiments, three separate animals for each condition were analyzed. (A,B,D) Immunohistochemistry on cross sections of 6 dpf wild-type and *Fip5b^CO40^* mutant larvae stained with Hoechst (blue), Phalloidin (red), and Rab11 (A), Rab7 (B), or Cytokeratin (D) (green). (C) Quantitation of Rab7-vesicle diameter. (E) Ratio of fluorescence intensity of apical keratin to cytoplasmic keratin. (F) Electron micrographs showing 11 dpf fed wild-type and *fip5b^CO40^* mutant larvae. Arrows point to larger than 500 nm organelles and brackets mark terminal web. (G) Quantitation of less than 500 nm apical vesicles in 11 dpf larvae. (H) Quantitation of greater than 500 nm organelles in 11 dpf larvae. (I) Quantitation of microvilli length in the intestinal bulb and midgut of 11 dpf larvae. Each dot represents a single microvillus length combined across three animals. All plots show mean±s.e.m. A *t*-test was used for Gaussian data and a Mann–Whitney test for all other statistics. ***P<0.0005, **P<0.005, *P<0.05.

To investigate the identity of the large organelles observed in *fip5b^CO40^* mutant larvae intestinal cells, we performed immunohistochemistry to detect proteins that serve as common endosome markers. Because Fip5 binds Rab11 vesicles, we first examined Rab11 localization. In wild-type intestinal cells, Rab11 vesicles localized just beneath the apical cell surface, as revealed by actin staining (Fig. 2A). In contrast, Rab11 vesicles mislocalized to the basolateral surface of intestinal cells in *fip5b^CO40^* mutant larvae (Fig. 2A). Because the large organelles observed through electron microscopy in mutant tissue were near the apical cell surface, they were unlikely to be Rab11-positive. Intestinal cells of MVID patients accumulate lysosomal granules (Iancu et al., 2007), so we next stained cells to detect the late endosome/lysosome marker Rab7. Notably, Rab7-positive organelles accumulated near the apical cell surface in *fip5b^CO40^* mutant cells, whereas we did not detect these large organelles in wild-type cells (Fig. 2B). These Rab7 endosomes were consistent in size and localization with the structures revealed by electron microscopy (Fig. 2C). Taken together, these data suggested that *fip5b* is required for Rab11 apical localization and regulation of trafficking processes.

Our electron microscopy analysis also revealed defects in microvilli length and the terminal web in *fip5b^CO40^* mutant cells. The terminal web is composed of actin and intermediate filaments and is located just below the apical cell surface to anchor the base of microvilli into the cell (Mooseker et al., 1984). Because actin localized to the apical cell surface of mutant cells similar to wild-type cells (Fig. 2A,B,D), we focused our attention on intermediate filaments. In polarized epithelia, intermediate filaments are composed of keratin polymers, so we stained cells with a pan-cytokeratin antibody to visualize intermediate filaments comprising the terminal web. We found that in wild-type cells, the keratin network resided just below the apical actin network; however, in *fip5b^CO40^* mutant cells, keratin mislocalized to lateral and cytoplasmic regions of the cell (Fig. 2D,E). These observations were consistent with the possibility that Fip5b regulates keratin polymerization and terminal web formation at the apical cell surface.

Terminal web defects result in microvilli abnormalities, which can be exacerbated by physical stress from intestinal activity. We therefore hypothesized that fed mutant larvae would show more severe microvilli phenotypes than unfed 6 dpf larvae still living off the yolk. To test this, we began feeding the larvae daily at 7 dpf and then analyzed larvae at 11 dpf. Mutant larvae showed moderate trafficking defects at 11 dpf (Fig. 2F, arrows, G,H); however, the terminal web defects recovered, and microvilli were now significantly longer than wild-type siblings (Fig. 2F, bracket, I). This phenotypic recovery was unexpected and perhaps explains in part why adult mutant fish were homozygous viable. Importantly, these trafficking and microvilli phenotypes were recapitulated in another *fip5b* mutant allele *fip5b^CO43^* (Figs S1B and S3A–D) indicating that these phenotypes were specific to *fip5b*. Taken together, these data provided evidence that *fip5b* functions in apical trafficking processes and microvilli formation during zebrafish intestinal development.

### *fip5a* functions similarly to *fib5b* in endosome maturation and terminal web organization

**Fig. 3.**
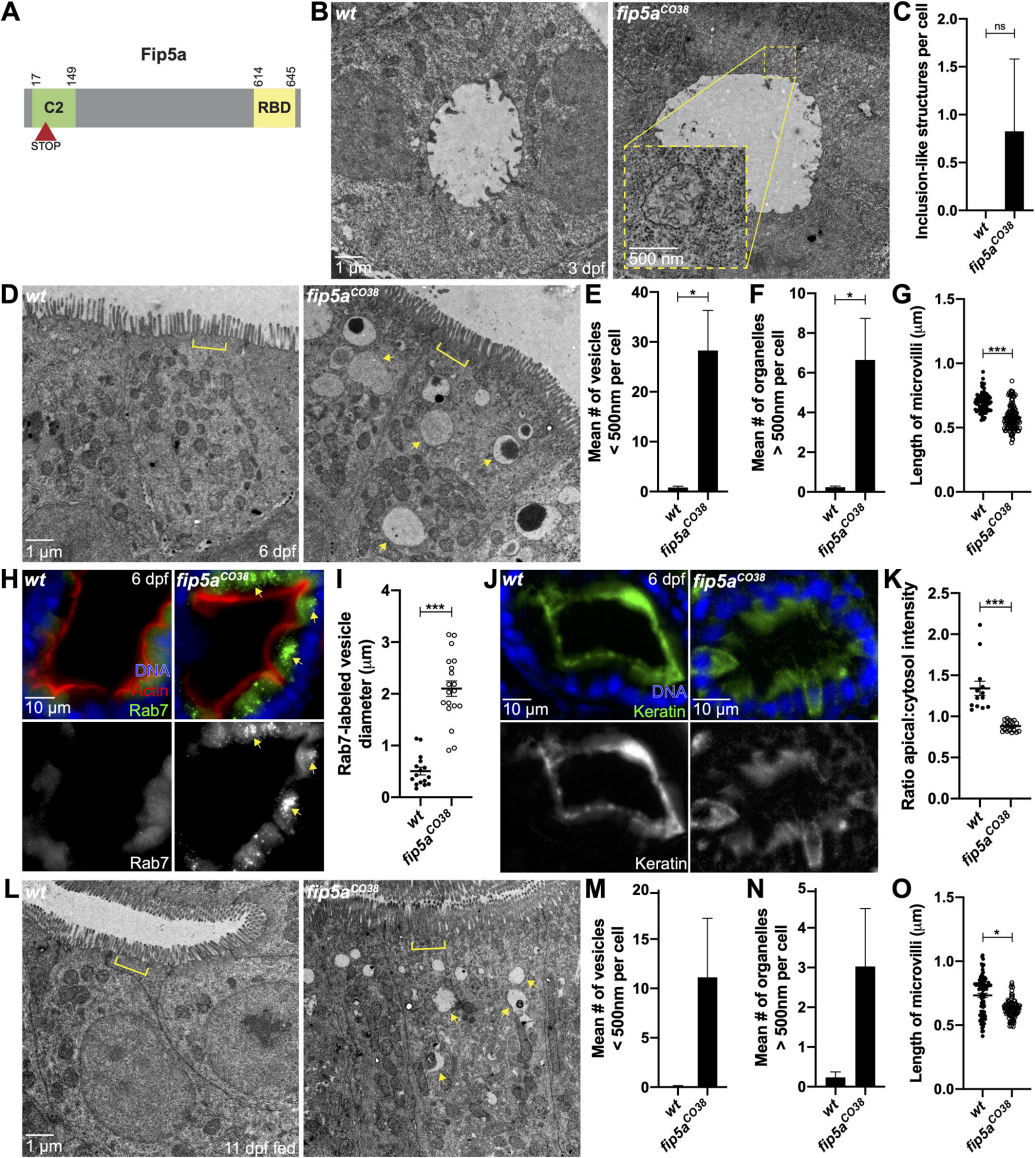
***fip5a* functions similarly to *fib5b* in endosome maturation and terminal web organization.** (A) Domain schematic of zebrafish Fip5a protein containing a C2 domain at the N-terminus and a Rab-binding domain (RBD) at the C-terminus. The red arrowhead with STOP denotes premature termination codon in *fip5a* mutant alleles. All following images are representative cross sections through midgut region. Wild-type siblings are used as controls. For all experiments, three separate animals for each condition were analyzed. (B) Electron micrographs showing 3 dpf wild-type and *fip5a^CO38^* mutant larvae. Yellow box shows zoomed in view on a region with subapical structures resembling microvillus inclusions. (C) Quantitation of the mean number of microvillus inclusion-like structures per cell in 3 dpf wild-type and *fip5a^CO38^* mutant larvae. (D) Electron micrographs showing 6 dpf wild-type and *fip5a^CO38^* mutant larvae. Arrows point to larger than 500 nm organelles and brackets mark terminal web or lack thereof in mutants. (E) Quantitation of less than 500 nm apical vesicles in 6 dpf larvae. (F) Quantitation of greater than 500 nm organelles in 6 dpf larvae. (G) Quantitation midgut microvilli length in 6 dpf larvae. Each dot represents a single microvillus length combined across three animals. (H) Immunohistochemistry on cross sections of 6 dpf wild-type and *fip5a^CO38^* mutant larvae stained with Hoechst (blue), Phalloidin (red), and Rab7 (green). (I) Quantitation of Rab7-vesicle diameter. (J) Immunohistochemistry on cross sections of 6 dpf wild-type and *fip5a^CO38^* mutant larvae stained with Hoechst (blue) and Cytokeratin (green). (K) Ratio of fluorescence intensity of apical keratin to cytoplasmic keratin. (L) Electron micrographs showing 11 dpf fed wild-type and *fip5a^CO38^* mutant larvae. Arrows point to larger than 500 nm organelles and brackets mark terminal web or lack thereof in mutants. (M) Quantitation of less than 500 nm apical vesicles in 11 dpf larvae. (N) Quantitation of greater than 500 nm organelles in 11 dpf larvae. (O) Quantitation midgut microvilli length in 11 dpf larvae. Each dot represents a single microvillus length combined across three animals. All plots show mean±s.e.m. A *t*-test was used for Gaussian data and a Mann–Whitney test for all other statistics. ***P<0.0005, *P<0.05.

Whereas *fip5b* mutant phenotypes were prominent during early developmental stages, these mutant fish recovered from these defects and were viable as homozygous adults. One possible explanation is a compensatory mechanism, perhaps through upregulation of another trafficking pathway, and an obvious candidate for compensation is the zebrafish *fip5b* paralog, *fip5a*. To test *fip5a*’s role in intestinal development, we again used CRISPR to create *fip5a*-mutant alleles (Fig. 3A, Fig. S1C). *fip5a*-mutant stocks were maintained in a heterozygous state and intercrossed to generate zygotic mutants for analysis. Stage matched wild-type siblings were used as controls. Similar to *fip5b* mutants, *fip5a^CO38^*-homozygous mutant larvae were morphologically normal and viable as homozygous adults. To study the role of *fip5a* during intestinal development, we performed the same transmission electron microscopy analysis on fixed sections through the mid-intestinal region. Notably, *fip5a^CO38^* mutant fish recapitulated phenotypes seen in *fip5b* mutant fish. At 3 dpf, *fip5a^CO38^* mutant larvae formed a lumen, but exhibited subapical organelles resembling microvillus inclusions (Fig. 3B,C). By 6 dpf, inclusion-like structures cleared, and *fip5a^CO38^* mutant cells now accumulated small apical vesicles (Fig. 3D,E) and large organelles (Fig. 3D, arrows, F) not present in wild-type larvae. Additionally, midgut microvilli were shorter (Fig. 3D,G) and the terminal web was also disrupted in mutants compared to wild-type larvae (Fig. 3D, brackets). Mutant larvae had large Rab7-positive organelles and terminal web defects appeared to be the result of mislocalized keratin from the apical cell surface (Fig. 3H–K). Again, similar to *fip5b* mutants at 11 dpf, *fip5a^CO38^* mutants maintained trafficking defects (Fig. 3L, arrows, M,N), but unlike *fip5b* mutants, the terminal web defects and shorter microvilli persisted in *fip5a^CO38^* mutants at 11 dpf (Fig. 3L, brackets, O). Importantly, these trafficking and microvilli phenotypes were recapitulated in another *fip5a* mutant allele, *fip5a^CO35^* (Figs S1C and S3E–H) indicating that these phenotypes were specific to *fip5a*. Collectively, these data implicated *fip5a* in apical trafficking and microvilli formation and suggested a similar function to *fip5b* during zebrafish intestinal development.

### *fip5a* and *fip5b* double mutants show severe microvilli and trafficking phenotypes

**Fig. 4.**
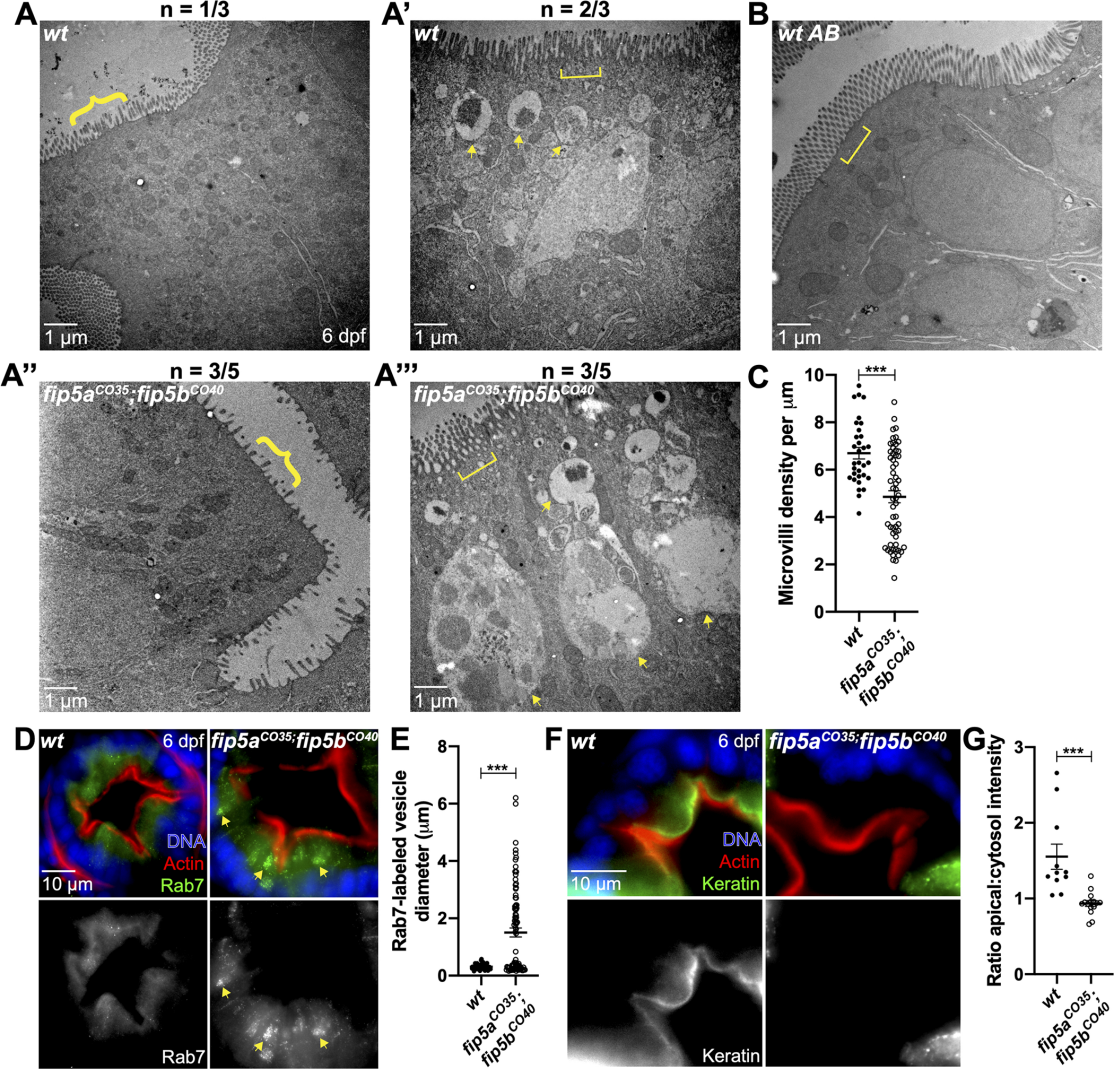
***fip5a* and *fip5b* double mutants show severe microvilli and trafficking phenotypes.** All following images are representative cross sections through the midgut region of 6 dpf larvae. (A–A′′′′) Electron micrographs showing wild-type siblings and *fip5a^CO35/CO35^; fip5b^CO40/CO40^* zygotic mutant larvae. Arrows point to larger than 500 nm organelles, braces point out sparse microvilli and brackets mark terminal web or lack thereof in mutants. N indicates number of representative larvae out of total number of larvae analyzed. (B) Electron micrograph showing wild-type AB larva. (C) Quantitation of microvilli density. Each dot represents the number of microvilli per micron for a field of view across three wild-type and five mutant animals. (D) Immunohistochemistry on cross sections of wild-type and *fip5a^CO35^; fip5b^CO40^* mutant larvae stained with Hoechst (blue), Phalloidin (red), and Rab7 (green). (E) Quantitation of Rab7-vesicle diameter. (F) Immunohistochemistry on cross sections of wild-type and *fip5a^CO35^; fip5b^CO40^* mutant larvae stained with Hoechst (blue), Phalloidin (red) and Cytokeratin (green). (G) Ratio of fluorescence intensity of apical keratin to cytoplasmic keratin. Three separate animals for each condition were analyzed. All plots show mean±s.e.m. A *t*-test was used for Gaussian data and a Mann–Whitney test for all other statistics. ***P<0.0005.

*fip5a*- and *fip5b*-homozygous mutant larvae showed similar phenotypes, but it remained unclear whether *fip5a* and *fip5b* function in parallel or through a common pathway. To test this, we created a *fip5a;fip5b* heterozygous mutant line (*fip5a^CO35/+^;fip5b^CO40/+^*). This fish line was maintained in a heterozygous state and intercrossed to generate *fip5a^CO35/CO35^;fip5b^CO40/CO40^* homozygous double-mutant embryos for experiments. Wild-type siblings were used as controls. Through electron microscopy analysis at 6 dpf, *fip5a^CO35^;fip5b^CO40^* zygotic double-mutant fish showed two classes of phenotypes. The first was a severe microvilli defect where microvilli density was significantly reduced and the microvilli that did form were shorter and more heterogeneous in double mutants compared to wild-type larvae (Fig. 4A and A″, braces, C). The second was a severe trafficking phenotype where the majority of the cell cytosol was filled with giant Rab7-positive organelles (Fig. 4A′ and A‴, arrows, D,E). Double-mutant fish also accumulated small apical vesicles and terminal web defects (Fig. 4A‴, bracket, F,G) like those seen in single mutants. These phenotypes were not mutually exclusive, as some mutant larvae displayed both microvilli and trafficking defects. It is worth noting that wild-type siblings also showed mild microvilli, terminal web and trafficking defects (Fig. 4A-A′, brace, bracket and arrows, respectively), perhaps suggestive of maternal contribution, as stage-matched wild-type AB fish did not show these phenotypes (Fig. 4B). In addition to these intestinal phenotypes, about 50% of the double-mutant larvae had multiple kidney lumens, whereas wild-type siblings or single *fip5a* or *fip5b* mutant larvae always had a single continuous kidney lumen (Fig. S3I). Moreover, double-mutant animals did not live past 2 weeks. Thus, the severity of these double-mutant phenotypes suggested that *fip5a* and *fip5b* function in parallel in microvilli formation during zebrafish intestinal development through apical trafficking pathways that regulate terminal web formation.

### Upregulation of Fip1 rescues *fip5a* and *fip5b* double-mutant phenotypes

**Fig. 5.**
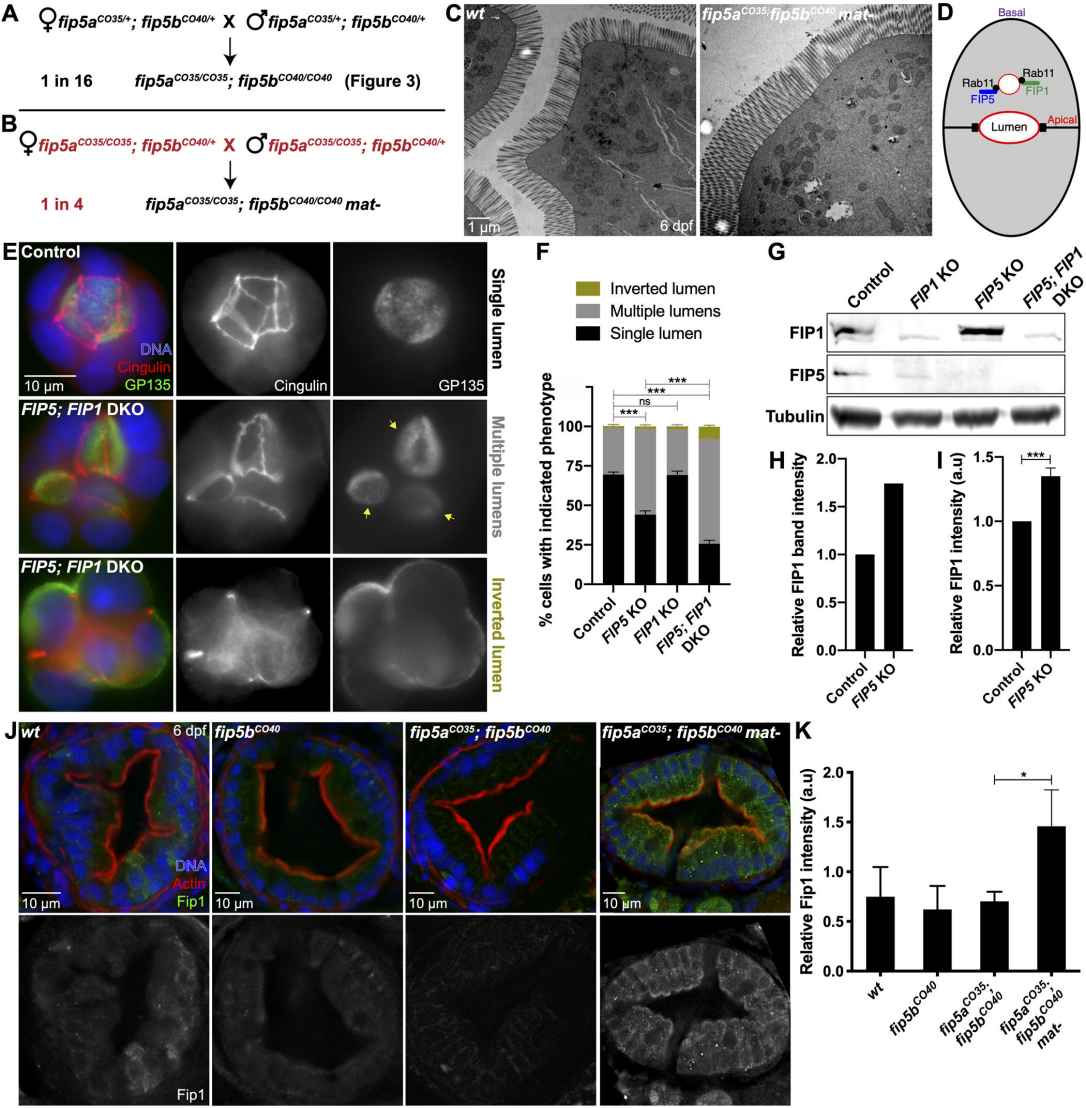
**Upregulation of Fip1 rescues *fip5a* and *fip5b* double-mutant phenotypes.** (A,B) Schematic of genetic crosses resulting in *fip5a^CO35^; fip5b^CO40^* double zygotic or maternal/zygotic (mat-) mutant offspring generated from two different parental genotypes. (C) Electron micrographs showing cross sections through midgut of 6 dpf wild-type AB and *fip5a^CO35^; fip5b^CO40^ mat-* mutant larvae. Three separate animals for each condition were analyzed. (D) Cartoon schematic showing FIP5 and FIP1 bind same Rab11 vesicles. (E) Wild-type and *FIP5; FIP1* double KO (DKO) MDCK cells grown in an extracellular matrix to induce 3D lumen formation. Arrows denote multiple lumens in KO cyst. (F) Quantitation of luminal phenotypes from three biological replicates. (G) Western blot on wild-type and KO MDCK cell lysates probed for FIP1, FIP5 or tubulin (control) antibodies. (H) Quantitation of FIP1 band intensity for wild-type and *FIP5* KO cell lysates from one biological replicate. (I) Quantitation of FIP1 fluorescence intensity in wild-type and *FIP5* KO cells grown in polarized monolayers from three biological replicates. Representative images are shown in Fig. S4A. (J) Immunohistochemistry on cross sections through midgut of 6 dpf wild-type, *fip5b^CO40^* mutant, *fip5a^CO35^; fip5b^CO40^* double mutant and *fip5a^CO35^; fip5b^CO40^ mat-* mutant larvae stained with Hoechst (blue), Phalloidin (red) and Fip1 (green). (K) Quantitation of fluorescence intensity of Fip1. Three separate animals for each condition were analyzed. All plots show mean±s.e.m. A *t*-test was used for Gaussian data and a Mann–Whitney test for all other statistics. ***P<0.0005, *P<0.05.

Although larvae deficient for zygotic functions of both *fip5a* and *fip5b* had severe intestinal phenotypes, contribution of wild-type maternal products to the eggs laid by heterozygous females potentially partially suppressed the phenotype. To test this possibility, we removed the maternal contribution of *fip5a* by intercrossing *fip5a^CO35/CO35^;fip5b^CO40/+^* adults. We called these maternal-zygotic double mutants *fip5a^CO35^;fip5b^CO40^ mat-* to differentiate from zygotic double mutants in Fig. 3 created from a heterozygous intercross (Fig. 5A versus B). Surprisingly, *fip5a^CO35^;fip5b^CO40^ mat-* larvae, lacking maternal and zygotic functions of *fip5a* and zygotic functions of *fip5b*, had no intestinal phenotypes and could not be discerned morphologically from wild-type larvae (Fig. 5C). Thus, removing maternal *fip5a* function suppressed, rather than enhanced, the phenotype of double-mutant larvae.

Recent literature has posited a role for compensatory mechanisms due to gene knockout when the mutant mRNA undergoes nonsense-mediated decay (Rossi et al., 2015; El-Brolosy et al., 2019). One compensatory mechanism included upregulation of transcripts similar in sequence to the mRNA encoded by the mutated gene (El-Brolosy et al., 2019). We thus wondered if another FIP family member could be upregulated in the absence of maternal and zygotic functions of *fip5a* and zygotic functions of *fip5b*. Previous work in our lab showed that both FIP5 and FIP1 bind the same Rab11 vesicles and FIP5 proteomics revealed an interaction with FIP1 (Willenborg et al., 2011; Mangan et al., 2016) (Fig. 5D). We thus used a MDCK tissue culture model of lumenogenesis to ask if FIP1 could compensate for FIP5. When MDCK cells were grown in an extracellular matrix, the majority of wild-type cells formed a single, continuous lumen inside the cyst of cells; however, most FIP5 and FIP1 double KO cells showed a multilumenal phenotype and a small percentage showed an inverted polarity phenotype (Fig. 5E,F). These luminal phenotypes were significantly more severe than FIP5 KO alone (Fig. 5F). Correspondingly, western blot analysis demonstrated that FIP1 protein levels were upregulated in FIP5 KO cells (Fig. 5G,H) and immunohistochemistry experiments with a FIP1 antibody confirmed this (Fig. 5I; Fig. S4A). This protein upregulation was specific to FIP1 in FIP5 KO cells, as FIP5 levels did not increase in FIP1 KO cells (Fig. 5G; Fig. S4B). Moreover, FIP5 and FIP1 double KO cells did not show general defects in apical polarity or tight junction formation when grown in a polarized monolayer (Fig. S4C,D), suggesting that FIP5 and FIP1 function were specific to apical trafficking during lumenogenesis.

Given that FIP1 could compensate for FIP5 in epithelial tissue culture, we asked if Fip1 could do the same *in vivo*. To test this, we performed immunohistochemistry on 6 dpf wild-type, *fip5b^CO40^*, *fip5a^CO35^;fip5b^CO40^*, and *fip5a^CO35^;fip5b^CO40^ mat-* larvae stained for endogenous Fip1 protein. Fip1 staining was mostly absent from wild-type, *fip5b^CO40^* mutant, and *fip5a^CO35^; fip5b^CO40^* zygotic double-mutant larvae; however, we observed a significant increase in Fip1 signal in *fip5a^CO35^;fip5b^CO40^ mat-* larvae, especially at the apical cell surface (Fig. 5J,K). This suggested that maternal contribution of wild-type *fip5a* may influence Fip1 protein levels to compensate for maternal and zygotic loss of Fip5a together with zygotic loss of Fip5b.

## DISCUSSION

Rab11 specificity for a particular cellular pathway is achieved through interacting with effector proteins, and our work revealed a role for the Rab11 effector paralogs Fip5a and Fip5b in apical trafficking and microvilli establishment during zebrafish intestinal development. In particular, we observed enlarged Rab7-positive, Rab11-negative organelles in mutants. Normally, there is a homeostasis established between Rab11 recycling from endosomes and maturation from early endosomes to lysosomes (Stenmark, 2009). We propose that without Rab11-Fip5-mediated removal and recycling of essential apical cargo, this homeostasis is disrupted such that cargo to be recycled builds up and the maturation process is delayed resulting in engorged Rab7-positive organelles. The exact identity of these organelles remains unclear. We speculate that they are lysosomes, but further work is needed to rule out the possibility of autophagosomes.

One characteristic of MVID is loss of microvilli at the apical cell surface, yet the mechanism behind microvilli phenotypes is still being revealed. Work from intestinal tissue culture and MYO5B mutant mice suggest that disruption of Rab11-mediated recycling of apical membrane proteins and transporters results in failure to maintain apical polarity (Knowles et al., 2014; Vogel et al., 2015; Weis et al., 2016). Our work posits an additional potential explanation in the intermediate filament networks. In polarized epithelia, groups of keratin proteins form polymers at the subapical cell surface, just below the apical actin cortex (Apodaca and Gallo, 2013). These actin and intermediate filament networks together comprise the terminal web, which is responsible for anchoring the microvilli rootlets into the cell. In *fip5*-mutant zebrafish, we observed loss of keratin localization from the apical cell surface to lateral and cytoplasmic regions. It remains unclear how Fip5 regulates apical keratin localization. Because keratins are cytosolic proteins whose assembly and disassembly into networks is mediated by phosphorylation state (Cooper, 2000), one possibility is that Rab11-Fip5 vesicles traffic a keratin kinase or phosphatase to the site of keratin polymerization thereby regulating network assembly. Alternatively, the effect of Fip5 on intermediate filament polymerization could be a more indirect result of general disruption in intracellular trafficking events as we see an accumulation of a number of vesicles and larger organelles in mutant cells. Because we used an endpoint analysis, we cannot fully differentiate whether Fip5 functions in the formation or maintenance of microvilli; however, our results demonstrate that without Fip5a or Fip5b, zebrafish intestinal microvilli are disrupted and major endosomal trafficking defects are observed in KO animals.

Mutations in MYO5B have been found in patients with MVID. As such, several MYO5B-deficient animal models have been developed to elucidate the cellular mechanism behind MVID. Our *fip5* mutant fish show a number of phenotypes consistent with these animal models including accumulation of apical vesicles, mislocalized Rab11, enlarged lysosomes, decreased microvilli length and structures resembling microvillus inclusions, although further work is need to fully demonstrate that these inclusion-like structures are equivalent to those in MYO5B-deficient animals. It is also interesting to note some differences. In zebrafish *myo5b* mutant larvae, microvillus inclusions are maintained until 6 dpf (Sidhaye et al., 2016), whereas in our mutants structures resembling inclusions were only present in 3 dpf larvae. This discrepancy could be due to differences in function between Myo5b and Fip5, with Myo5b regulating more diverse trafficking pathways than Fip5. Consistent with this, studies in tissue culture have shown that MYO5B binds FIP2, another Rab11 effector closely related to FIP5 (Hales et al., 2002). Alternatively, our data show that the zebrafish paralogs Fip5a and Fip5b function in parallel, whereas fish only contain one gene for Myo5b, potentially causing *myo5b* mutants to demonstrate stronger phenotypes than single *fip5a* or *fip5b* mutants. Moreover, loss of microvillus inclusions has been observed in MYO5B mutant mice, where inclusions were more pronounced in neonates and disappeared after weaning (Knowles et al., 2014; Weis et al., 2016).

Our analyses of zygotic and maternal/zygotic double-mutant animals raise interesting questions about gene compensation and maternal contribution. Whereas zygotic double-mutant larvae showed severe trafficking and microvilli defects and did not survive past two weeks, it was surprising that the wild-type siblings also exhibited a milder form of these phenotypes because age-matched wild-type AB larvae did not demonstrate any phenotypes. This led us to hypothesize that maternal contribution of *fip5a* and *fip5b*, even in heterozygotes, are likely important for proper intestinal development. In support of this, full maternal depletion of *fip5a* triggers protein upregulation of the closely related family member Fip1. Future studies, such as measuring maternal RNA levels of *fip5a* and *fip5b*, will be needed to confirm the putative involvement of maternal contribution on intestinal development. Furthermore, the mechanism behind increased Fip1 protein levels remains unclear. Recent literature has demonstrated a role for nonsense-mediated decay in genetic compensation (Rossi et al., 2015; El-Brolosy et al., 2019), and this explanation is consistent with our data suggesting nonsense-mediated decay in *fip5b* mutants. In conclusion, our work implicates the Rab11 effectors Fip5 and Fip1 in apical trafficking and microvilli formation during zebrafish intestinal development.

## MATERIALS AND METHODS

### Antibodies

The following antibodies were used in this study: Pan cytokeratin AE1/AE3 (Abcam/ab27988) 1:50, Rab7 (Abcam/ab50533) 1:100, Rab11 (Life Technologies/715300) 1:100, GP135 (DSHB/3F2/D8) 1:100, Cingulin (Prekeris Lab) 1:100, FIP1 (Prekeris Lab) 1:200, FIP5 [Prekeris Lab (1:100)], Alexa 488 Anti-Rabbit secondary (Jackson ImmunoResearch/711-545-152) 1:200, Alexa 488 Anti-Mouse secondary (Jackson ImmunoResearch/715-545-150) 1:200, Alexa-568 Phalloidin (Invitrogen/A12380) 1:100.

### Zebrafish husbandry

All animal work was approved by the Institutional Animal Care and Use Committee at the University of Colorado School of Medicine, CO, USA. All stocks unless otherwise specified were maintained in a heterozygous state and kept according to Standard Operating Procedure defined in ‘The Zebrafish Book’ (M. Westerfield, Institute of Neuroscience, University of Oregon, OR, USA).

### qRT-PCR

RNA extraction from larvae was performed with TRIzol reagent (Invitrogen) followed by cDNA synthesis with iScript cDNA Synthesis Kit (Bio-Rad). SYBR Green PCR Master Mix (Applied Biosystems) was used for qPCR. All reactions were performed in technical triplicate and a minimum of three biological replicates were performed. Primer sequences are listed in Table S1.

### Protein alignments

Fip5 protein alignments were generated using T-Coffee and Boxshade 3.2. The following protein accession numbers from NCBI were used for alignments: human NP_056285; dog XP_003639656 (isoform X5); zebrafish Fip5a XP_009305489 (isoform X2); zebrafish Fip5b XP_017214658 (rab11 family-interacting protein 5-like isoform X2).

### Zebrafish immunohistochemistry

At all timepoints analyzed, animals had not yet developed sex organs, thus experiments were blinded to gender. Larvae were placed in 1–2% Tricaine for 10 min or until they were unresponsive to touch then decapitated immediately posterior to the otic vesicle using a scalpel. The larva body was placed in fix solution (4% paraformaldehyde, 4% sucrose, 0.15 mM CaCl_2_, pH 7.3) at 4°C overnight, whereas the head was placed in lysis buffer and genotyped (see genotyping). The fixed larvae were then embedded in a melted agar solution (1.5% agar, 5% sucrose in water) and, after the blocks hardened, they were trimmed and immersed in 30% sucrose in water solution overnight at 4°C. Blocks were then dried with a chemwipe, frozen on dry ice for ∼15 min, then stored at -80°C until ready to section.

Blocks were mounted in OCT and 20 μm sections cut using a Leica CM 1950 cryostat microtome. Sections were placed on FisherBrand charged slides (12-550-15) and rehydrated in PBS for 30 min. Excess liquid was dried, and then a wax pen was used to draw around the edge of the slide. Slides were then blocked with 2% BSA and 5% donkey serum (Thermo Fisher Scientific, NC9624464) in PBS for 1 h, then incubated in primary antibody diluted in block at room temperature for 2–3 h. Slides were then washed four times, for 15 min each with PBS and incubated in secondary antibody diluted in block for 1–2 h at room temperature. Slides were again washed four times, for 15 min each, with PBS, adding Hoescht (Thermo Fisher Scientific, 33342 at 1:500) to the second-to-last wash. Slides were then dried, mounted in Vectashield (Vector Laboratories, H-100), and sealed with nail polish.

### Widefield microscopy and image analysis

All slides of fixed fish sections were imaged with an inverted Axiovert 200M microscope (Carl Zeiss) with a 63× oil immersion lens and QE charge-coupled device camera (Sensicam). Images were acquired using Slidebook 6.0 (Intelligent Imaging Innovations) software. Images were processed using a combination of Slidebook 6.0 (Intelligent Imaging Innovations) software, Fiji (PMID 22743772), and Adobe Photoshop. Figures were made in Adobe Illustrator. A minimum of three biological replicates were performed for each experiment and quantitation was performed unblinded.

### Genotyping zebrafish

Fish tissue was isolated from a fin clip for adult fish or from the heads for larvae. Fish tissue was placed in lysis buffer (10 mM Tris pH 8.3, 50 mM KCl, 1.5 mM MgCl_2_, 0.3% Tween-20, 0.3% NP-40 in water) with 2% Proteinase K (Invitrogen, 25530049). Lysis reactions were incubated at 55°C for 4 h, then 95°C for 20 min to inactivate Proteinase K. A PCR/restriction-enzyme-based assay was used to genotype *fip5a* and *fip5b* mutant fish lines. For *fip5a*, a 400 base pair region of genomic DNA surrounding the CRISPR target site was amplified by PCR. The PCR product was then digested with BssHII for 1 h at 50°C, and the resulting product was run on a 2% agarose gel. For *fip5b*, a similar schematic was used with the BsaWI or AgeI restriction enzyme depending on the allele. PCR primer sequences are listed in Table S1. Genotyping was performed prior to experiments and only wild-type and homozygous mutant larvae were selected for analysis.

### CRISPR/Cas9 in zebrafish

All primer sequences are listed in Table S1. Guide RNA (gRNA) oligos were designed using ZiFIT Targeter Software for CRISPR/Cas9 Nucleases (Sander et al., 2010, 2007). The gRNA target sites were then blasted (NCBI Blastn) against the zebrafish genome to look for potential off target sites. gRNA oligos were annealed and phosphorylated, then cloned into the *pDR274* vector (Addgene) using the BsaI-HF restriction site. Positive clones were sequenced to confirm correct insertion. The gRNA-containing vector was linearized using DraI and purified by ethanol precipitation. The gRNA sequence was then transcribed to RNA using T7 polymerase and purified by phenol choloroform extraction. Cas9 mRNA was synthesized from the pT3TS vector (Addgene) using mMESSAGE mMACHINE T3 Transcription Kit (Thermo Fisher Scientific, AM1348) and purified using phenol choloroform extraction. The injection mix was prepared as follows: 0.2 M potassium chloride, 0.15 ng μl^−1^ Cas9 mRNA, 70 ng μl^−1^ gRNA, and 10% phenol red in DEPC water. Embryos were injected with 1–3 nl of the injection mixture at the one-cell stage. Founder fish were determined using T7E1 analysis (NEB), and positive hits were sequenced to determine exact mutation. Founders from two different gRNA injection experiments containing different mutant alleles for *fip5a* and *fip5b* were selected and outcrossed for at least two generations before performing experiments.

### Transmission electron microscopy

Larvae were placed in 1–2% Tricaine for 10 min or until they were unresponsive to touch then decapitated immediately posterior to the otic vesicle. The larva body was placed in EM fix solution (0.1 M sodium cacodylate, 4% paraformaldehyde, 4% glutaraldehyde, in PBS) at 4°C overnight, whereas the head was placed in lysis buffer and genotyped. The body was processed for EM by washing in 0.1 M sodium cacodylate, then incubating tissue in 500 μl of 1:1 osmium tetroxide to 0.1 M sodium cacodylate for 2 h. Tissue was washed with double-distilled water, then incubated in 500 μl 1:1 osmium tetroxide to imidazole (0.35 g imidazole in 25 ml sodium cacodylate pH to 7.4) for 30 min. Larvae turned brown at this point. Larvae were washed again in double-distilled water, then an ethanol dehydration series was performed (50%/75%/100%). Larvae were then incubated in 1:1 Epon to ethanol for 1 h, then 2:1 Epon to ethanol overnight. The following day, larvae were embedded in 100% Epon, which was replaced with fresh 100% Epon, and then baked for 2 days. Larvae were cut in half where the body narrows (see schematic in Fig. 1C), and then 65 nm thick sections were cut and collected on formvar-coated copper slot grids. Sections cut in the anterior direction were designated the intestinal bulb region and in the posterior direction the midgut. Sections were imaged on a FEI Tecnai G2 Biotwin Transmission Electron Microscope, run at 80 kV with a side-mount AMT XR80S-B digital camera. For transmission electron microscopy quantitation, a minimum of three biological replicates were used for each experiment and images were blinded prior to analysis.

### RNA *in situ* hybridization

Sense and antisense RNA probes were designed against both the coding sequence and 3′ UTR region of zebrafish *fip5a* and *fip5b* genes. A PCR-based method with T7 sites at the end of the primers was used to amplify the probe DNA sequence from 8 dpf wild-type fish cDNA (see Table S1). The RNA probes were transcribed with the T7 polymerase and labeled using the DIG RNA labeling kit (Roche, 11175025910). After the labeling reaction was complete, the probes were mixed with 1 μl 0.5 M EDTA to stop the reaction, then 2 μl 5 M lithium chloride and 75 μl cold ethanol were added for purification by ethanol precipitation and the probe was resuspended in DEPC water. The RNA probe was checked by agarose gel electrophoresis, then mixed with an equal volume of formamide and stored at -80°C.

RNA *in situ* hybridization assays were conducted based on a modified version of a previously published protocol described by Hauptmann and Gerster (2000). Larvae were fixed in 4% paraformaldehyde in DEPC PBS overnight at 4°C. Larvae were stored in MeOH at –20°C until use, when they were washed twice for five minutes in DEPC-PBSTw (0.5% Tween-20 in PBS made with DEPC water). Pigmentation was bleached in a hydrogen peroxide solution (3% H_2_O_2_, 0.5% KOH in DEPC water) until larvae eyes turned brown (15–30 min). Larvae were then washed twice for 5 min in DEPC-PBSTw, fixed for 20 min at room temperature in 4% PFA in DEPC-PBS, and washed again twice for 5 min in DEPC-PBSTw. The larvae were digested with 0.1 mg ml^−1^ Proteinase K (Invitrogen, 25530049) in DEPC-PBS for 17 min to permeabilize the larvae, then washed twice for 5 min each wash in DEPC-PBSTw, followed by fixation for 20 min in 4% PFA, and again washed twice for 5 min in DEPC-PBSTw. Larvae were incubated in 500 μl hybridization media (HM) block solution (50% formamide, 5× saline-sodium citrate buffer, 10 μl ml^−1^ tRNA, 50 mg ml^−1^ heparin, 0.01 M citric acid and 0.5% Tween-20 in DEPC H_2_O) for 1 h at 70°C. The block was replaced with HM containing 200 ng of the appropriate RNA probe, and the larvae were incubated at 70°C overnight. The following day, a series of progressive washes were performed for 10 min each wash at 70°C: 200 μl 100% HM without probe, 300 μl 66% HM/33% 2× saline-sodium citrate buffer (SSC; Cellgro, Mediatech, Inc., Manassas, VA, USA), 300 μl 33% HM/66% 2× SSC, 1 ml 2× SSC, 1 ml 0.2× SSC, 1 ml 0.1× SSC (this wash was performed twice), 1 ml DEPC-PBSTw. Another 10 min wash with DEPC-PBSTw was performed at room temperature, followed by a 1 h antibody block (2% sheep serum and 2 mg ml^−1^ BSA in DEPC-PBSTw). Anti-digoxigenin-AP Fab fragments (Roche Applied Science, Indianapolis, IN, USA) was incubated in antibody block overnight at 4°C. Finally, four 15 min washes were conducted at room temperature in DEPC-PBSTw. Larvae were incubated in staining solution (0.1 M Tris, pH 9.5, 0.25 M MgCl_2_, 0.1 M NaCl, 0.5% Tween-20) for 15 min at room temperature. Thereafter, the larvae were moved to a staining dish, covered with 500 μl precipitating BM Purple AP Substrate (Roche Applied Science, Indianapolis, IN, USA), and incubated at 37°C for 8 h until staining was visible. The larvae were then washed twice for 5 min in PBS and imaged or processed for sectioning immediately.

### Cell culture and immunohistochemistry

MDCK II cells (ATCC and negative for mycoplasma contamination) were cultured in DMEM with 10% FBS and penicillin/streptomycin. For polarized MDCK experiments, cells were plated on collagen type I-coated Transwell filters (Corning, 3460) to reach confluency in 24 h. Cells were then grown for a further 3 days before transepithelial resistance measurements or fixation. Cells were fixed with 4% paraformaldehyde for 20 min at room temperature. Cells were blocked for 1–2 h in block buffer (PBS, 0.1% Triton X-100, 10% normal donkey serum). Primary antibodies were diluted in block buffer and incubated overnight at room temperature. Cells were washed with PBSTx before adding secondary antibodies for 1–2 h at room temperature. Cells were washed again before mounting in VectaShield and sealing with nail polish or mounting in Prolong Gold. Coverslips used for all experiments were #1 thickness.

### Trans-epithelial resistance measurements

MDCK cells were grown on collagen-coated Transwell filters (see Cell culture and immunohistochemistry section) and resistance measurements were taken four days after plating with a Millicell ERS-2 Voltohmmeter (Millipore). Three measurements per well, one for each space between the plastic prongs of the filter holder, were averaged and subtracted from the average of the blank well containing a collagen-coated filter without any cells.

### Generating MDCK and RPE-1 CRISPR knockout lines

MDCK cells stably expressing Tet-inducible Cas9 (Dharmacon Edit-R inducible lentiviral Cas9 nuclease) were grown in a 12-well dish to about 75% confluency before treatment with doxycycline at a final concentration of 1 μg ml^−1^ for 24 h to induce Cas9 expression. Cells were then transfected with crRNA:tracrRNA mix as described for DharmaFECT Duo co-transfection protocol (Horizon Discovery, T-2010-xx). Transfected cells were incubated for 24 h before trypsinizing and plating for individual clones. Individual clones were screened through genotyping PCR and Sanger sequencing. All CRISPR gRNAs are listed in Table S1.

## Supplementary Material

Supplementary information

## References

[BIO055822C1] Al-Daraji W. I., Zelger B., Zelger B. and Hussein M. R. (2010). Microvillous inclusion disease: a clinicopathologic study of 17 cases from the UK. *Ultrastruct. Pathol.* 34, 327-332. 10.3109/01913123.2010.500447 21070163

[BIO055822C2] Alvers A. L., Ryan S., Scherz P. J., Huisken J. and Bagnat M. (2014). Single continuous lumen formation in the zebrafish gut is mediated by smoothened-dependent tissue remodeling. *Development* 141, 1110-1119. 10.1242/dev.100313 24504339 PMC3929411

[BIO055822C3] Apodaca G. and Gallo L. I. (2013). Epithelial polarity. In *Colloquium Series on Building Blocks of the Cell: Cell Structure and Function* (ed. Nabi I. R.). Morgan & Claypool Life Sciences.

[BIO055822C4] Cooper G. M. (2000). Intermediate Filaments.

[BIO055822C5] EL-Brolosy M. A., Kontarakis Z., Rossi A., Kuenne C., Günther S., Fukuda N., Kikhi K., Boezio G. L. M., Takacs C. M., Lai S.-L. et al. (2019). Genetic compensation triggered by mutant mRNA degradation. *Nature* 568, 193-197. 10.1038/s41586-019-1064-z 30944477 PMC6707827

[BIO055822C6] Hales C. M., Vaerman J.-P. and Goldenring J. R. (2002). Rab11 family interacting protein 2 associates with Myosin Vb and regulates plasma membrane recycling. *J. Biol. Chem.* 277, 50415-50421. 10.1074/jbc.M209270200. 12393859

[BIO055822C29] Hauptmann G. and Gerster T. (2000). Multicolor whole-mount *in situ* hybridization. Methods *Mol. Biol*. **137**, 139-148. 10.1385/1-59259-066-7:139 PMID: 10948532.

[BIO055822C7] Horgan C. P. and Mccaffrey M. W. (2009). The dynamic Rab11-FIPs. *Biochem. Soc. Trans.* 37, 1032-1036. 10.1042/BST0371032 19754446

[BIO055822C8] Iancu T. C., Mahajnah M., Manov I. and Shaoul R. (2007). Microvillous inclusion disease: ultrastructural variability. *Ultrastruct. Pathol.* 31, 173-188. 10.1080/01913120701350712 17613997

[BIO055822C9] Jewett C. E. and Prekeris R. (2018). Insane in the apical membrane: trafficking events mediating apicobasal epithelial polarity during tube morphogenesis. *Traffic* 19, 666-678. 10.1111/tra.12579 PMC6239989 29766620

[BIO055822C10] Knowles B. C., Roland J. T., Krishnan M., Tyska M. J., Lapierre L. A., Dickman P. S., Goldenring J. R. and Shub M. D. (2014). Myosin Vb uncoupling from RAB8A and RAB11A elicits microvillus inclusion disease. *J. Clin. Invest.* 124, 2947-2962. 10.1172/JCI71651 24892806 PMC4071383

[BIO055822C11] Lapierre L. A., Kumar R., Hales C. M., Navarre J., Bhartur S. G., Burnette J. O., Provance D. W., Mercer J. A., Bähler M. and Goldenring J. R. (2001). Myosin Vb is associated with plasma membrane recycling systems. *Mol. Biol. Cell* 12, 1843-1857. 10.1091/mbc.12.6.1843 11408590 PMC37346

[BIO055822C12] Mangan A. J., Sietsema D. V., Li D., Moore J. K., Citi S. and Prekeris R. (2016). Cingulin and actin mediate midbody-dependent apical lumen formation during polarization of epithelial cells. *Nat. Commun.* 7, 12426 10.1038/ncomms12426 27484926 PMC4976216

[BIO055822C13] Mooseker M. S., Bonder E. M., Conzelman K. A., Fishkind D. J., Howe C. L. and Keller T. C. (1984). Brush border cytoskeleton and integration of cellular functions. *J. Cell Biol.* 99, 104s-112s. 10.1083/jcb.99.1.104s 6378918 PMC2275581

[BIO055822C14] Müller T., Hess M. W., Schiefermeier N., Pfaller K., Ebner H. L., Heinz-Erian P., Ponstingl H., Partsch J., Röllinghoff B., Köhler H. et al. (2008). MYO5B mutations cause microvillus inclusion disease and disrupt epithelial cell polarity. *Nat. Genet.* 40, 1163-1165. 10.1038/ng.225 18724368

[BIO055822C15] Ng A. N. Y., de Jong-Curtain T. A., Mawdsley D. J., White S. J., Shin J., Appel B., Dong P. D. S., Stainier D. Y. R. and Heath J. K. (2005). Formation of the digestive system in zebrafish: III. Intestinal epithelium morphogenesis. *Dev. Biol.* 286, 114-135. 10.1016/j.ydbio.2005.07.013 16125164

[BIO055822C16] Phillips A. D. and Schmitz J. (1992). Familial microvillous atrophy: a clinicopathological survey of 23 cases. *J. Pediatr. Gastroenterol. Nutr.* 14, 380-396. 10.1097/00005176-199205000-00003 1355534

[BIO055822C17] Phillips A. D., Jenkins P., Raafat F. and Walker-Smith J. A. (1985). Congenital microvillous atrophy: specific diagnostic features. *Arch. Dis. Child.* 60, 135-140. 10.1136/adc.60.2.135 3977385 PMC1777158

[BIO055822C18] Prekeris R., Davies J. M. and Scheller R. H. (2001). Identification of a novel Rab11/25 binding domain present in Eferin and Rip proteins. *J. Biol. Chem.* 276, 38966-38970. 10.1074/jbc.M106133200 11481332

[BIO055822C19] Rossi A., Kontarakis Z., Gerri C., Nolte H., Hölper S., Krüger M. and Stainier D. Y. R. (2015). Genetic compensation induced by deleterious mutations but not gene knockdowns. *Nature* 524, 230-233. 10.1038/nature14580 26168398

[BIO055822C20] Ruemmele F. M., Schmitz J. and Goulet O. (2006). Microvillous inclusion disease (microvillous atrophy). *Orphanet J. Rare Dis.* 1, 22 10.1186/1750-1172-1-22 16800870 PMC1523325

[BIO055822C21] Ruemmele F. M., Müller T., Schiefermeier N., Ebner H. L., Lechner S., Pfaller K., Thöni C. E., Goulet O., Lacaille F., Schmitz J. et al. (2010). Loss-of-function of MYO5B is the main cause of microvillus inclusion disease: 15 novel mutations and a CaCo-2 RNAi cell model. *Hum. Mutat.* 31, 544-551. 10.1002/humu.21224 20186687

[BIO055822C22] Sander J. D., Zaback P., Joung J. K., Voytas D. F. and Dobbs D. (2007). Zinc Finger Targeter (ZiFiT): an engineered zinc finger/target site design tool. *Nucleic Acids Res.* 35, W599-W605. 10.1093/nar/gkm349 17526515 PMC1933188

[BIO055822C23] Sander J. D., Maeder M. L., Reyon D., Voytas D. F., Joung J. K. and Dobbs D. (2010). ZiFiT (Zinc Finger Targeter): an updated zinc finger engineering tool. *Nucleic Acids Res.* 38, W462-W468. 10.1093/nar/gkq319 20435679 PMC2896148

[BIO055822C24] Sidhaye J., Pinto C. S., Dharap S., Jacob T., Bhargava S. and Sonawane M. (2016). The zebrafish goosepimples/myosin Vb mutant exhibits cellular attributes of human microvillus inclusion disease. *Mech. Dev.* 142, 62-74. 10.1016/j.mod.2016.08.001 27497746 PMC5161235

[BIO055822C25] Stenmark H. (2009). Rab GTPases as coordinators of vesicle traffic. *Nat. Rev. Mol. Cell Biol.* 10, 513-525. 10.1038/nrm2728 19603039

[BIO055822C26] Vogel G. F., Klee K. M. C., Janecke A. R., Müller T., Hess M. W. and Huber L. A. (2015). Cargo-selective apical exocytosis in epithelial cells is conducted by Myo5b, Slp4a, Vamp7, and Syntaxin 3. *J. Cell Biol.* 211, 587-604. 10.1083/jcb.201506112 26553929 PMC4639860

[BIO055822C27] Weis V. G., Knowles B. C., Choi E., Goldstein A. E., Williams J. A., Manning E. H., Roland J. T., Lapierre L. A. and Goldenring J. R. (2016). Loss of MYO5B in mice recapitulates Microvillus Inclusion Disease and reveals an apical trafficking pathway distinct to neonatal duodenum. *Cell. Mol. Gastroenterol. Hepatol.* 2, 131-157. 10.1016/j.jcmgh.2015.11.009 27019864 PMC4806369

[BIO055822C28] Willenborg C., Jing J., Wu C., Matern H., Schaack J., Burden J. and Prekeris R. (2011). Interaction between FIP5 and SNX18 regulates epithelial lumen formation. *J. Cell Biol.* 195, 71-86. 10.1083/jcb.201011112 21969467 PMC3187708

