# Supplementary material for: The Rab11 effectors Fip5 and Fip1 regulate zebrafish intestinal development
Source: Biol Open. 2020 Oct 23;9(10):bio055822. doi: 10.1242/bio.055822 (PMC7595698; doi:10.1242/bio.055822)
Supplement: Supplementary information [file biolopen-9-055822-s1.pdf]

Biology Open (2020): doi:10.1242/bio.055822: Supplementary information

A

```
HumanFIP5         1 MALVRGAEPAAGPSRWLPTHVQVTVLRARGLRGKSSGAGSTSDAYTVIQVGREKYSTSVV
DogFIP5           1 MALVRGAEPAPGPSRWLPTHVQVTVLRARGLRGKSSGAGSTSDAYTVIQVGREKYSTSVV
ZebrafishFip5a    1 MSLAKS-D---EDQRWVPTHVQVTVLRARGLRAKG--KHGTSDVYTIIQLGKEKYSTCVM
ZebrafishFip5b    1 MPLISLDD---EEQRWVPTHVNVTVLRARALRTKG--KQGSRYVYTIIQVGKEKYTTGLV

HumanFIP5        61 EKTHGCPEWREECSFELPPGALDGLLRAQEADAGPAPWAASSAAACELVLTTMHRSLIGV
DogFIP5          61 EKTPGCPEWREECSFELPPGALDGLLRAQEADAGSAPWAAGSAAACELVLTTMHRSLIGV
ZebrafishFip5a   55 EKTT-DPEWGEECSFELQPGILEEEGR--------DAY---PPGSGDLTLTVMHRALIGL
ZebrafishFip5b   56 EKAE-EPQWGEECAFELLPGLLEAGGT--------SAY---PPGSSNLVFTVMHRVLIGL

HumanFIP5       121 DKFLGQATVALDEVFGAGRAQHTQWYKLHSKPGKKEKERGEIEVTIQFTRNNLSASMFDL
DogFIP5         121 DKFLGQATVALDEVFGAGRAQHTQWYKLHSKAGKKEKERGEIQVTIQFTRNNLSASMFDL
ZebrafishFip5a  103 DVFLGQAVLPLHKAFQDRKSKKNEWHRLHSKTGKKEKERGELQLSVQFTRHNLTASMYDL
ZebrafishFip5b  104 DVFLGQTIVPLDKVFQEGTCPRNEWLKLHSKAGRKEKERGELQVTIQFTRNNMTASMYDL

HumanFIP5       181 SMKDKPRSPFSKIRDKMKGKKKY-DLESASAILPSSA-------------------I-ED
DogFIP5         181 SMKDKPRSPFSKIKDKMKGKKKF-DLESASAILPSSA-------------------L-ED
ZebrafishFip5a  163 SMKDKPRSAFDKLRERMRAKKRPAEEDSSSAIVPGGYGALARMRGRLP-SDGGGEEDYED
ZebrafishFip5b  164 TVKDKPRSAFGKLKDRVTGKKRD--VESSSAVLPGRYAALSGSVGPPFAGDGGSYEA-SE

HumanFIP5       220 PDLGSLGKMGKAKGFFLRNKLRKSSLTQSNTSLGSDSTLSSASGSLAYQGPGA----ELL
DogFIP5         220 PELGSLGKMGKAKGFFLRNKLRKSSLTQSNTSLGSDSTLSSASGSLAYQGPGT----ELL
ZebrafishFip5a  222 -DEGGEARRSKMRSFFLRGRLRKSSDTRSSTSLGSESSESSSRGGSLSPTAGISVVVSDL
ZebrafishFip5b  221 -EDGVEEHRSKVKDFFLKGKLRKNSDTRSCSSLASDSSMASSAGDPFIPV--------EI

HumanFIP5       276 TRSPSRSSWLSTEGGRDSAQSPK--LFTHKRTYSDEANQMRVAPPRALLDLQGHLDA---
DogFIP5         276 THSPSRSSWLSTEGGRDSTQSPK--LLTHKRTYSDEASQMRVAPPRSLLDLQGHLDA---
ZebrafishFip5a  281 SNSPSNSSNLTADNSPEHTVAPSPQVSPVRHVMYD----ISLPVPHS------MMSDNDT
ZebrafishFip5b  272 PRTPIYSSRVMEPFRMDTEEAIK--VMTHKRAHSDEASKITCVPRPS-----PAVEN---

HumanFIP5       331 -ASRSSLCVNGSHIYNEEPQGPVR---------HRSSISGSL------------------
DogFIP5         331 -ASRSSLCVNGSHIYNEEPQAPLR---------HRSSISGPF------------------
ZebrafishFip5a  331 PILLPSVCVNGNPVETS----PLTHHPPTLVLQH-PQQESTKPITQSGQPQATKLPAKPE
ZebrafishFip5b  322 -LSQSTLCINGSHIYSSEPVSPKSP---SAIPAKRSLLEKCA------------------

HumanFIP5       363 ----------------------PSSGSLQAVSSRFSEEGPR---STDDTWPRGSRSNSSS
DogFIP5         363 ----------------------PPSSSLHSVSFRPAEEGSR---PTDDSGGRGSRSTSSS
ZebrafishFip5a  386 KSQESKPRPEPRLPALGVLQKGSLSLSLQNLSRQ-GKEKQNGGPVD---GRRWSFDKPGE
ZebrafishFip5b  360 ----------------------PLSRSLQNLTRR-GEDSQK---SD---GRRWSIDKSKK

HumanFIP5       398 EAVLGQEELSAQAKVLAPGASHPGEEEGARLPEGKPVQV--ATPIVASSEAVAEKEGARK
DogFIP5         398 EMLPGQEELSSQAKVLATGTSRSGEEEGARLPEGKPVQV--ATPLVASSESVAEKEGARK
ZebrafishFip5a  442 E-------------EKAAIVA-ALEHAGR--VTDEPVNETVIRAG------------ETE
ZebrafishFip5b  391 E-------------DLETNAA-QSQTQGSTIVDGKPVQA--AGAVD-----------VLD

HumanFIP5       456 EERKPRMGLFHHHHQGLSRSELGRRSSLGEKGGPIL-GASPHHSSSGEEKAKSSWFGLRE
DogFIP5         456 EERKPRMGLFHHHHQGLSRSELGRRGSLGEKGGPTQ-GASPHHSSGGEEKAKSSWFGLRE
ZebrafishFip5a  474 TQGKKRRGLFSH----------GKGDSAGKG--PITSKEETEHAQPLVEVKHKGWFSS--
ZebrafishFip5b  424 KGKKLRKTLFSS----------GRSDSLPAK--P-----EQGQVSAPVEGRRRGWFGS--

HumanFIP5       515 AKDPTQKPSPHPVKPLSAAPVEGSPDRKQSRSSLSIALSSGLEKLKTV-TSGSIQPVTQA
DogFIP5         515 AKEPTQKPSPHPVKPLSAASLEGSPDKKQSRSSLSTALSSGLEKLKTV-TSGSVQPVAPA
ZebrafishFip5a  520 -KDSHSKPSPHPVKPLTPPDE----KRSEGR--------SVLEKLKSTIHSGRSDA----
ZebrafishFip5b  465 -GDSQNKPSPHPVKPLTNNTLQGE-KKAESR--------SVLEKLKSTINPGRSALATTA

HumanFIP5       574 PQAGQMVDTKRLKDSAVLDQSAKYYHLTHDELISLLLQRERELSQRDE------------
DogFIP5         574 PHVGQTVDTKRLKDSGVLDQSAKYYHLTHDELISLLLQRERELSQRDE------------
ZebrafishFip5a  563 ------------DKKPLVEGGGSYYHLNHSELVNLLIQRDMELRQEREEYEKRGMLLEKR
ZebrafishFip5b  515 EEE--------KQQLSLMEARAHYQNMTNMELIALLLQQELEIKKQRAETEVQVVMLEKR

HumanFIP5       622 ---------HVQELESYIDRLLVRIMETSPTLLQIPPGPPK
DogFIP5         622 ---------HVQELESYIDRLLVRIMETSPTLLQIPPDPPK
ZebrafishFip5a  611 ETDLKKMKLLIKDLEDYIDTLLVRIMEQTPTLLQVRPK-MK
ZebrafishFip5b  567 DAELKKMKVQVRDLEDYIDKLLVRIMEQTPTLLQVRGR-LK
```

B <u>**Fip5b Exon 2**</u>

WT Fip5b: WLKLHSKAGRKEKERGELQVTIQFTRNNMTASMYDLTVKDKPRSAFGKLKDRVTGKKRDVESSSAVLPGRYA ALSGSVGPPFAGDGGSYEASEEDGVEEHRSKVKDFFLKGKLRKNSDTRSCSSLASD

Fip5b^{CO40}: ALSGSVGPPFAGDGGSYEASEEDGVEEHGVEEVRSKTSFStop

Fip5b^{CO43}: ALSGSVGPPFAGDGGSYEASEEDGVEEHRSKTSFStop

C <u>**Fip5a Exon 1**</u>

WT Fip5a: MSLAKSDEDQRWVPTHVQVTVLRARGLRAKGKHGTSDVYTIIQLGKEKYSTCVMEKTTDPEWGEECSFEL

Fip5a^{CO35}: MSLAKSDEDQRWVPTHVQVTVLRARGLREGQTRHQRRVHHHPAGQGEILHVRDGEDYRSGMGRGMLVStop

Fip5a^{CO38}: MSLAKSDEDQRWVPTHVQVTVLRARGLRVTStop

Biology Open • Supplementary information

Figure S1. (A) Protein alignments for human FIP5, dog FIP5, and zebrafish paralogs Fip5a and Fip5b. The yellow highlighted region denotes the C2 domain and the blue highlighted region denotes the Rab-binding domain. (B) Fip5b exon 2 sequence in wild-type, *fip5b*^{CO40} mutant, and *fip5b*^{CO43} mutant alleles. Red amino acids show where mutants differ from wild-type allele. (C) Fip5a exon 1 sequence in wild-type, *fip5a*^{CO35} mutant, and *fip5a*^{CO38} mutant alleles. Red amino acids show where mutants differ from wild-type allele.

Biology Open • Supplementary information

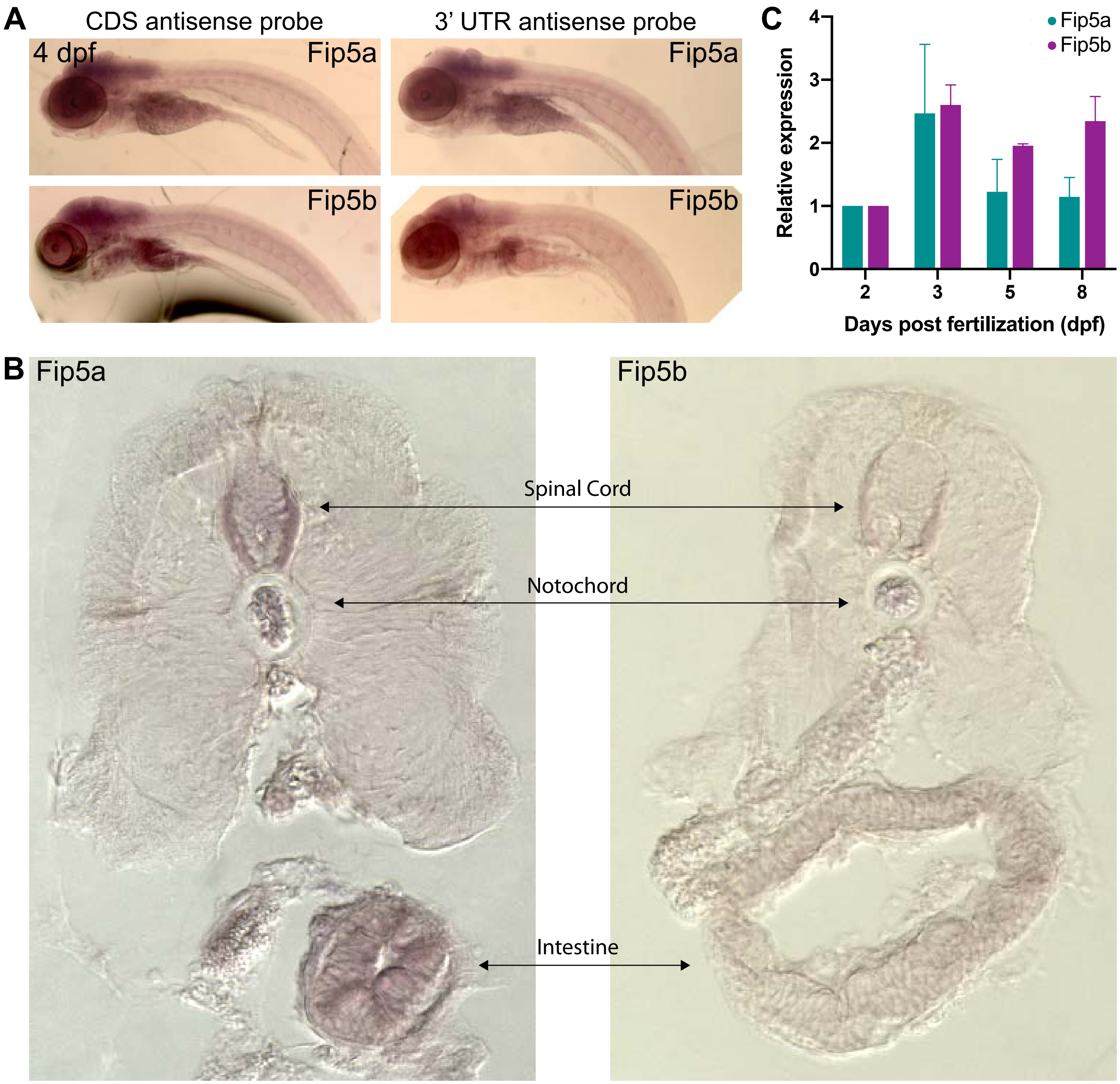

Figure S2. *In situ* hybridization on 4 dpf larvae with antisense probes for the coding sequences of *fip5a* and *fip5b* (left panel) and the 3' UTR sequences of *fip5a* and *fip5b* (right panel). (B) Representative cross sections of *fip5a* and *fip5b* antisense coding sequence probes. (C) qRT-PCR measuring *fip5a* and *fip5b* transcript levels at 2, 3, 5, and 8 dpf normalized to levels at 2 dpf. Three independent experiments were performed. All plots show mean ± s.e.m.

Biology Open • Supplementary information

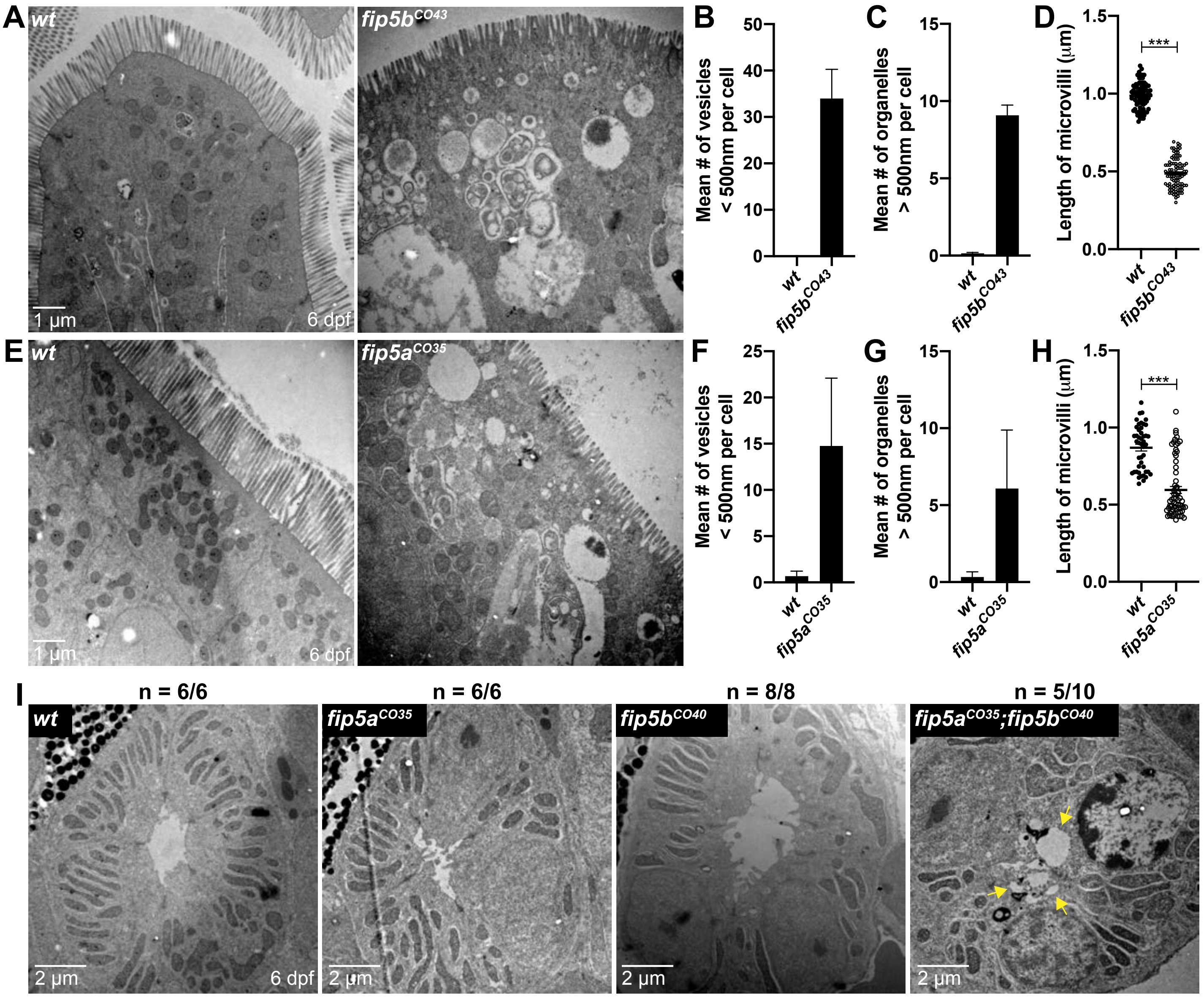

Figure S3. All following images are representative cross sections through midgut region on 6 dpf larvae. Wild-type siblings are used as controls. For A-G, three separate animals for each condition were analyzed. (A) Electron micrographs showing wild-type and *fip5b*^{CO43} mutant larvae. (B) Quantitation of less than 500nm apical vesicles. (C) Quantitation of greater than 500nm organelles. (D) Quantitation of midgut microvilli length. Each dot represents a single microvillus length combined across three animals. (E) Electron micrographs showing wild-type and *fip5a*^{CO35} mutant larvae. (F) Quantitation of less than 500nm apical vesicles. (G) Quantitation of greater than 500nm organelles. (H) Quantitation of midgut microvilli length. Each dot represents a single microvillus length combined across three animals. (I) Electron micrographs of kidneys in wild-type, *fip5b*^{CO40} mutant, *fip5a*^{CO35} mutant, and *fip5a*^{CO35}; *fip5b*^{CO40} double mutant larvae. N indicates number of representative kidneys out of total number of kidneys analyzed. Arrows point to multiple lumens in *fip5a*^{CO35}; *fip5b*^{CO40} double mutant larvae. All plots show mean ± s.e.m. A t-test was used for Gaussian data and a Mann-Whitney test for all other statistics. ***P < 0.0005.

Biology Open • Supplementary information

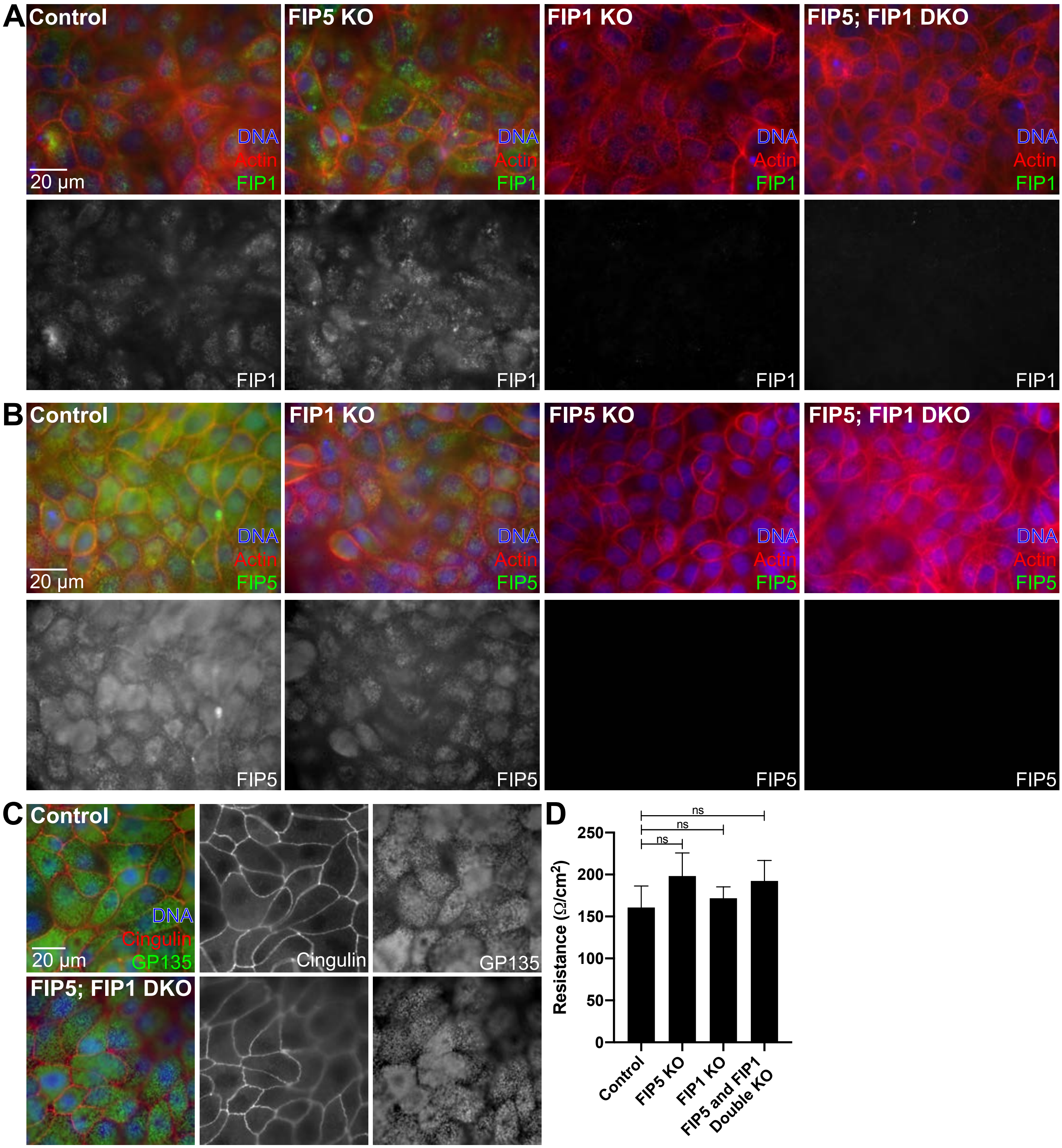

Figure S4. (A) Wild-type, FIP5 KO, FIP1 KO, and FIP5 and FIP1 double KO MDCK cells grown in polarized monolayers and stained for Hoechst (blue), Phalloidin (red) and Fip1 (green). (B) Wild-type, FIP1 KO, FIP5 KO, and FIP5 and FIP1 double KO MDCK cells grown in polarized monolayers and stained for Hoechst (blue), Phalloidin (red) and Fip5 (green). (C) Wild-type and FIP5 and FIP1 double KO MDCK cells grown in polarized monolayers and stained for Hoechst (blue), the tight junction marker Cingulin (red) and the apical membrane marker GP135 (green). (D) Trans-epithelial resistance measurements on wild-type, FIP5 KO, FIP1 KO, and FIP5 and FIP1 double KO MDCK cells grown in polarized monolayers from three biological replicates.

Table S1. Primer sequences. Primers were designed using the NCBI/Primer-BLAST tool.

Fip5a ISH For Antisense	TACAACAAACGCCTCCGCTA
Fip5a ISH Rev Antisense	TAATACGACTCACTATAGGGCGCGTTGTGCAACAAAAACC
Fip5b ISH For Antisense	GAAGCGCTCCGTCCCAAATA
Fip5b ISH Rev Antisense	TAATACGACTCACTATAGGGTGATTCACTACAATCTCAGACCTCA
Fip5a genotyping For	CCACTGTCTTATGTGCCCGT
Fip5a genotyping Rev	TGCTCTTCCGATCCTGAAAGG
Fip5b genotyping For	GAGAGCTACAGGTCACCATCC
Fip5b genotyping Rev	GCTGTAAATCGGTGTTCTGGG
Fip5aExon1gRNAolig1	TAGGCCCGAGGGTTGCGCGCGA
Fip5aExon1gRNAolig2	AAACTCGCGCGCAACCCTCGGG
Fip5bExon2gRNAolig1	TAGGTGGAAGAACACCGGAGTA
Fip5bExon2gRNAolig2	AAACTACTCCGGTGTTCTTCCA
FIP5Bset1For qPCR	GGCAAACTATTGTTCCGCTCG
FIP5Bset1Rev qPCR	TTGTTGCGGGTGAACTGGAT
FIP5Bset2For qPCR	AAATCCAGGACGATCTGCTCT
FIP5Bset2Rev qPCR	CGCTGCTTCTTGATCTCCAAT
Rpl13aFor qPCR	TCTGGAGGACTGTAAGAGGTATGC
Rpl13aRev qPCR	AGACGCACAATCTTGAGAGCAG
GAPDHFor qPCR	GTGGAGTCTACTGGTGTCTTC
GAPDHRev qPCR	GTGCAGGAGGCATTGCTTACA
FIP1KO gRNA	GTGATAACCCAAGGGCACTG
FIP5KO gRNA	GGGTTCATTTGGGGTCACAT

Biology Open • Supplementary information
